# Molecular Cloning and Effects of *Tm14-3-3ζ*-Silencing on Larval Survivability Against *E. coli* and *C. albicans* in *Tenebrio molitor*

**DOI:** 10.3390/genes9070330

**Published:** 2018-06-29

**Authors:** Jeong Hwan Seong, Yong Hun Jo, Gi Won Seo, Soyi Park, Ki Beom Park, Jun Ho Cho, Hye Jin Ko, Chang Eun Kim, Bharat Bhusan Patnaik, Sung Ah Jun, Yong Seok Choi, Young Wook Kim, In Seok Bang, Yong Seok Lee, Yeon Soo Han

**Affiliations:** 1 Division of Plant Biotechnology, Institute of Environmentally-Friendly Agriculture (IEFA), College of Agriculture and Life Sciences, Chonnam National University, Gwangju, 61186, Korea; dmdtkal222@nate.com (J.H.S.); yhun1228@jnu.ac.kr (Y.H.J.); ndnd2@nate.com (G.W.S.); soyipark@gmail.com (S.P.); misson112@naver.com (K.B.P.); junhojo12@naver.com (J.H.C.); hjngo0129@naver.com (H.J.K.); chang9278@naver.com (C.E.K.); 2 School of Biotech Sciences, Trident Academy of Creative Technology (TACT), Chandrasekharpur, Bhubaneswar, Odisha, 751024, India; drbharatbhusan4@gmail.com; 3 Institute of Medical Science, University of Toronto, Toronto, ON, M5S 1A8, Canada; s.jun@mail.utoronto.ca; 4 Department of Hotel Food Service and Culinary Arts, Seowon University, Cheongju, 28674, Korea; trues67@naver.com; 5 Korean Edible Insect Laboratory, Joong-gu, Shindang-dong, Seoul, 04598, Korea; kei_lab@naver.com; 6 Department of Biological Science, Hoseo University, Asan, 31499, Korea; isbang@hoseo.edu; 7 Department of Life Science and Biotechnology, College of Natural Sciences, Soonchunhyang University, Asan, 31538, Korea; yslee@sch.ac.kr

**Keywords:** *Tenebrio molitor*, 14-3-3ζ, molecular cloning, peptide-based antibody, mortality, RNA interference

## Abstract

The 14-3-3 family of proteins performs key regulatory functions in phosphorylation-dependent signaling pathways including cell survival and proliferation, apoptosis, regulation of chromatin structure and autophagy. In this study, the zeta isoform of 14-3-3 proteins (designated as Tm14-3-3ζ) was identified from the expressed sequence tags (ESTs) and RNA sequencing (RNA-Seq) database of the coleopteran pest, *Tenebrio molitor*. *Tm14-3-3ζ* messenger RNA (mRNA) is expressed at higher levels in the immune organs of the larval and adult stages of the insect and exhibit almost five-fold induction within 3 h post-infection of the larvae with *Escherichia coli* and *Candida albicans*. To investigate the biological function of Tm14-3-3ζ, a peptide-based Tm14-3-3ζ polyclonal antibody was generated in rabbit and the specificity was confirmed using Western blot analysis. Immunostaining and confocal microscopic analyses indicate that Tm14-3-3ζ is mainly expressed in the membranes of midgut epithelial cells, the nuclei of fat body and the cytosol of hemocytes. Gene silencing of *Tm14-3-3ζ* increases mortality of the larvae at 7 days post-infection with *E. coli* and *C. albicans*. Our findings demonstrate that 14-3-3ζ in *T. molitor* is essential in the host defense mechanisms against bacteria and fungi.

## 1. Introduction

The 14-3-3 family constitutes a highly conserved group of eukaryotic proteins that play a pivotal role in the regulation of cell survival, apoptosis and signal transduction [1,2,3]. They bind to a functionally wide array of cellular proteins that include kinases, phosphatases, receptors, structural proteins and transcription factors [4,5,6]. In fact, over 200 target ligands of 14-3-3 proteins are known and the binding of 14-3-3 proteins to their ligands is facilitated by the consensus phosphoserine or threonine containing 14-3-3 binding motifs in the amino acid sequence of the protein [7,8]. The binding, in turn, affects interaction of the ligand with other proteins, largely due to the conformational alteration of the ligand [9]. Previous reports show that a deletion of 14-3-3 isoform results in loss of viability in yeast [7,10]. Similarly, loss of *Drosophila* 14-3-3ζ leads to impaired viability of the embryo even in the presence of a functional 14-3-3ε isoform [11]. Studies in mammalian models of disease have shown the association of 14-3-3ζ proteins with epilepsy development and other neurological conditions such as seizures [12,13].

Since their initial discovery, 14-3-3 family members have been reported from both vertebrates and invertebrates. The number of 14-3-3 encoding genes varies, with only one gene reported from the lower eukaryote, *Candida albicans* [10], seven genes encoding seven distinct isoforms (β, ε, γ, η, ζ, θ and σ) from most mammals and up to thirteen genes in *Arabidopsis thaliana* [14]. A pair of 14-3-3 genes (isoforms ζ and ε), have been identified in insects including *Drosophila melanogaster* [15], *Bombyx mori* [16] and *Anopheles* mosquitoes [17,18]. The 14-3-3ζ protein shows a ubiquitous expression pattern in the tissues during the developmental stages of *B. mori* [19,20]. Recently, a *14-3-3ζ* homolog has been characterized from the Indian meal moth, *Plodia interpunctella*, a very common household pest feeding principally on stored food products [21]. Earlier studies have focused on the role of 14-3-3ζ in the regulation of immune signaling in insects such as *Antheraea pernyi*, *B. mori*, *Heliothis virescens*, *Spodoptera litura* and *D. melanogaster* [22,23,24]. It has also been suggested that 14-3-3ζ is required in the regulation of developmental autophagy and diapause processes in insects [25].

The coleopteran insect—*Tenebrio molitor—*has been extensively studied for immune genes and signaling mechanisms including the extracellular and intracellular components of Toll signaling cascades [26,27,28,29], immune deficiency (imd) pathway [30], other regulatory genes such as *CD63* [31], *R-type lectin* [32], *apolipophorin-III* [33], *14-3-3 epsilon* [34] and autophagy signaling genes [35,36]. In the present study, we identified the ζ isoform of 14-3-3 gene (*Tm14-3-3ζ*) from *T. molitor* expressed sequence tag (EST) and RNA sequencing database and characterized the full-length complementary DNA (cDNA) and protein sequence using in silico tools. Furthermore, we generated a peptide-based Tm14-3-3ζ polyclonal antiserum from rabbit and confirmed its specificity with the recombinant Tm14-3-3ζ protein (rTm14-3-3ζ). We used the antiserum to analyze the subcellular localization of Tm14-3-3ζ in *T. molitor* larvae. We then analyzed the putative role of Tm14-3-3ζ in innate immune response by silencing the *Tm14-3-3ζ* transcript in the larvae of the host insect by exogenous double-stranded RNA (dsRNA) treatment. Taken together, our results demonstrated the requirement of Tm14-3-3ζ in innate immune response against *Escherichia coli* and *C. albicans* in *T. molitor*.

## 2. Materials and Methods

### 2.1. Insects

*Tenebrio molitor* larvae were collected from Pusan National University, Pusan, South Korea and reared on an artificial diet (4.4 g NeoVita, 0.5 g Chloramphenicol, 0.4 g Ascorbic Acid, 0.5 g Sorbic Acid, 0.5 mL Propionic Acid, 2.2 g Yeast dry powder, 2.2 g Bean powder, 7.6 g Agar, 4.4 g Wheat powder, 73.3 g Wheat bran, in 200 mL of distilled water) sterilized at 121 °C for 15 min. The reared larvae were maintained at 26 ± 1 °C and 60 ± 5% relative humidity in dark conditions.

### 2.2. Microbial Cultures and Bioassays

The *E. coli* K12 and *C. albicans* cultures were used in the infection and bioassay experiments. Luria-Bertani (LB) broth (BD Biosciences, Franklin Lakes, NJ, USA) and Sabouraud Dextrose broth (BD Biosciences) were used to culture *E. coli* and *C. albicans*, respectively. For all experiments, overnight cultures at 37 °C were harvested, washed twice and re-suspended in phosphate buffered saline medium (1X PBS; 130 mM NaCl, 7 mM Na_2_HPO_4_, 3 mM NaH_2_PO_4_·H_2_O; pH 7.2) by centrifugation (Hanil science co., Ltd, Daejeon City, Korea) at 3500 rpm for 10 min. The cultures were serially diluted to attain a final concentration of 10^9^ colony-forming unit (CFU)/ml and 5 × 10^7^ CFU/ml for *E. coli* and *C. albicans*, respectively. 

For immune challenge experiments, 1 µL of culture suspension equivalent to 10^6^ CFU/Larva for *E. coli* or 5 × 10^4^ CFU/Larva for *C. albicans* were microinjected into healthy *T. molitor* larvae using a Picospritzer III micro-dispense system (Parker, Hollis, NH, USA). PBS (1 µL volume) was injected into a separate group of *T. molitor* larvae as an injection control. 

### 2.3. Isolation of Tm14-3-3ζ Full-Length Complementary DNA Sequence

A partial cDNA sequence of *Tm14-3-3ζ* was screened from *T. molitor* EST and RNA sequencing (RNA-Seq) database. For the amplification of full-length cDNA sequence of *Tm14-3-3ζ*, gene-specific oligonucleotides were designed using Primer 3.0 software (http://bioinfo.ut.ee/primer3-0.4.0/) (Table 1). The 5′- and 3′-RACE-ready cDNAs were synthesized from 1 µg of total RNA using SMARTer rapid amplification of cDNA ends (RACE) amplification kit (Clontech Laboratories, CA, USA) according to manufacturer’s instructions. Real time polymerase chain reaction (RT-PCR) was performed at 94 °C for 30 s (denaturation), 55 °C for 30 s (annealing) and 72 °C for 30 s (extension) for 30 cycles. The gene-specific primer and the nested primer sequences have been enlisted in Table 1. The PCR products were purified using the AccuPrep PCR purification kit (Bioneer, Daejeon, Korea), cloned into TOPO TA cloning vector (Invitrogen Corporation, Carlsbad, CA, USA) and subsequently transformed into the competent *E. coli* (DH5α strain) cells. 

### 2.4. Protein Domain and Phylogenetic Analysis

The structural domains of Tm14-3-3ζ were predicted using InterProScan (https://www.ebi.ac.uk/interpro/search/sequence-search) and Simple modular Architecture research tool (SMART) version 7.0) [37]. Using the SMART screen page, protein family (PFAM) domains, signal peptides and internal repeats were validated. The amino acid sequences of 14-3-3ζ proteins from various insect species were retrieved from National Center for Biotechnology Information (NCBI) database (https://www.ncbi.nlm.nih.gov/). Multiple sequence alignment (MSA) and percentage identity analysis were done using ClustalX software [38]. The distance analysis and the construction of phylogenetic tree were performed using the maximum-likelihood method (JTT matrix model) using the MEGA 6 software [39]. The GenBank accession numbers of 14-3-3ζ amino acid sequences are provided in Table S1.

### 2.5. Quantitative Polymerase Chain Reaction Analysis

Relative quantification of mRNA transcripts was conducted by RT-PCR and analyzed by the comparative C_T_ method. Total RNAs were isolated from the different developmental stages and larval/adult tissues of the insect using the TRIZOL reagent method (Favorgen Biotech Corp., Pingtung County, Taiwan). Then cDNAs were synthesized with 2 µg of total RNAs using AccuPower^®^ RT Pre-Mix (Bioneer) and oligo (dT)_12–18_ primer on a C1000 Touch PCR machine (Bio-Rad, USA). Signals were detected using the gene-specific primers listed in Table 1 and AccuPower^®^ 2X green star quantitative PCR (qPCR) master mix (Bioneer) by Exicycler 96 RT-PCR machine (Bioneer). PCR conditions were as follows: An initial denaturation of 95 °C for 20 s followed by 40 cycles at 95 °C for 5 s and 60 °C for 20 s. 60S ribosomal protein *L27a* gene from *T. molitor* (*TmL27a*) was used as an internal control.

### 2.6. Generation of Peptide-Based Tm14-3-3ζ Antiserum and Reactivity Studies

A 16-mer peptide (N-SDTQGEADEPQETGDN-C) from the C-terminal part of Tm14-3-3ζ was designed and synthesized (Ab Frontier Co. Ltd., Seoul, Korea). A cysteine residue was added to the C-terminus of the peptide for conjugation to carrier protein. The peptide was conjugated to Keyhole Limpet Hemocyanin (KLH) and bovine serum albumin (BSA) (Sigma, St. Louis, MO, USA) separately, using Maleimidobenzoyl-N-hydroxysuccinimide ester (MBS). The peptide-KLH was used for immunization of rabbit and peptide-BSA was used for testing the specificity of the antibody.

The specificity of the peptide-based Tm14-3-3ζ antiserum was examined with the whole-body lysate of last instar *T. molitor* larvae and the bacterial lysate induced by isopropyl β-D-1- thiogalactopyranoside (IPTG) using Western blot analysis. Samples were homogenized in 1X phosphate buffered saline (PBS, pH 7.4) and centrifuged at 13,000× *g* for 10 min at 4 °C to remove the cell debris. The total protein concentration in the supernatant fraction was determined using the Bradford dye-binding method. Proteins were separated in 15% sodium dodecyl sulfate-polyacrylamide gel electrophoresis (SDS-PAGE) system and transferred onto a polyvinylidendifluoride (PVDF) membrane. The PVDF membrane was blocked for 1 h in 5% non-fat milk in Tris-buffered saline (TBS) containing 0.1% Tween 20 (TBST; 10 mM Tris-HCl, 150 mM NaCl, 0.1% Tween 20, pH 7.5), followed by an incubation of 3 h with anti-Tm14-3-3ζ rabbit serum (1:5000 in blocking buffer). The membrane was then washed six times for 10 min each in large volumes of wash buffer (TBST). The washed membrane was incubated in alkaline-phosphatase-conjugated secondary antibody (Sigma-Aldrich, St. Louis, Missouri, USA) for 1 h and visualized using a nitro blue tetrazolium/5-bromo-4-chloro-3-indolyl-phosphate (NBT/BCIP) solution. Anti-His-monoclonal antibody (Applied Biological Materials Inc., Richmond, British Columbia, Canada) was used as a positive control to detect the His-tagged rTm14-3-3ζ.

### 2.7. Subcellular Localization of Tm14-3-3ζ by Immunofluorescence

The tissues from the last-instar larvae of *T. molitor* such as gut, fat body, Malpighian tubules and hemocytes were fixed in 4% paraformaldehyde in 1X PBS, pH 7.4 for 12 h at 4 °C. The fixed tissues were rinsed in 1X PBS, followed by incubation in a sucrose gradient (12–20% sucrose in PBS). Samples were embedded into FSC22 clear resin (Leica Biosystems, Wetzlar, Germany) and cryosectioned with Leica CM1850 cryostat (Leica Biosystems). The cryosections were blocked for 1 h with 2% BSA in 1X PBS containing 0.2% Tween 20 (PBST). Subsequently, the sections were incubated in Tm14-3-3ζ antiserum (diluted 1:300 in blocking buffer) for 3 h. After three washes (5 min each) with PBST, cells were incubated for 1 h with Alexa Flour 488 dye-conjugated secondary antibody (1:300 dilution in PBST). The nucleus and the actin molecules were detected by TO-PRO-3 iodide (1:300 dilution in PBST) and Alexa Flour 568 Phalloidin (1:300 dilution in PBST), respectively. The samples were mounted with Dako Cytomation fluorescent mounting medium (Dako, Carpentaria, CA, USA). Sub-cellular localization of Tm14-3-3ζ was recorded using a Fluoview 500 Confocal microscopic system (Olympus, Ina, Japan).

### 2.8. Double Stranded RNA Treatment and Gene Silencing

To synthesize dsRNA for *Tm14-3-3ζ*, specific primers conjugated with T7 promoter sequences were designed using the Snapdragon dsRNA design software [40,41]. The primer sequences are available in Table 1. Enhanced green fluorescent protein (EGFP) dsRNA was synthesized from pEGFP-C1 plasmid vector (Clontech Laboratories, CA, USA) and injected as the negative control. Template cDNAs for ds*Tm14-3-3ζ* synthesis were amplified by using ExTaq polymerase (Takara, kusatsu, Japan) with specific primers as described in Table 1. PCR products containing the T7 promoter sequences were purified using the AccuPrep PCR purification kit (Bioneer). ds*Tm14-3-3ζ* was synthesized with 1 µg of purified PCR products using the Ampliscribe T7-flash transcription kit (Epicentre Biotechnologies, Madison, Wisconsin, USA). Synthesized dsRNAs were confirmed by electrophoresis on 1% agarose gels. For gene specific silencing, 1 µg of ds*Tm14-3-3ζ* were injected into *T. molitor* larvae using Picospritzer III micro-dispense system (Parker). ds*EGFP* was injected as a negative control using the same concentration. Transcript levels of *Tm14-3-3ζ* were analyzed using real-time PCR.

### 2.9. Mortality Assay

The *E. coli* (1 × 10^6^ cells per larva) or *C. albicans* (5 × 10^4^ cells per larva) were injected into ds*Tm14-3-3ζ*-treated *T. molitor* larvae. Mortality was monitored for seven days and expressed as percentage of dead larva per day per treatment. ds*EGFP* injected group for each pathogen was used as a negative control. Each injection group consisted of ten larvae and the experiment was conducted in triplicates.

## 3. Results

### 3.1. Cloning and Sequence Analysis of Tm14-3-3ζ Gene

**Figure 1:**
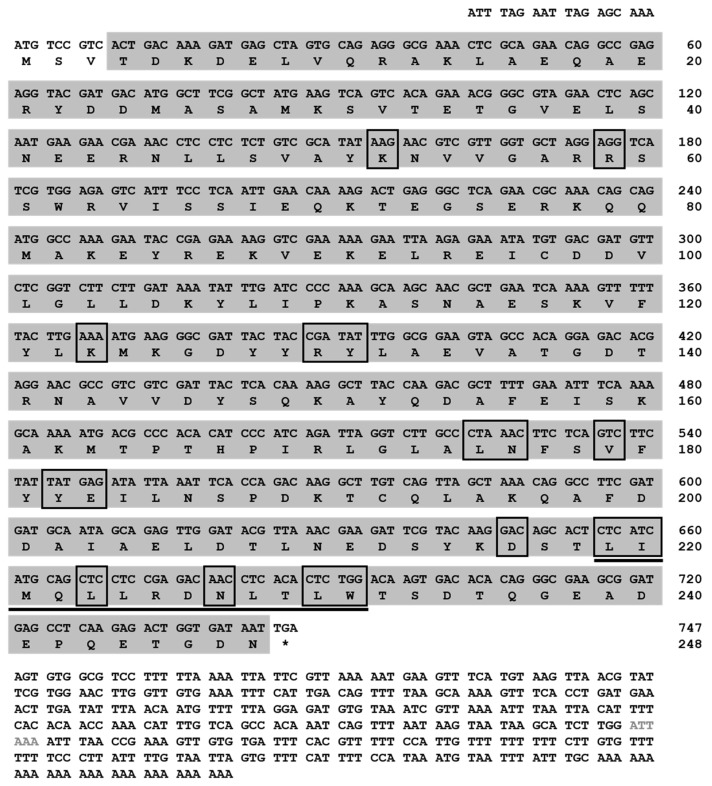
The nucleotide and deduced amino acid sequence of Tm14-3-3*ζ*. The deduced protein sequence is shown below the nucleotide sequence. The complementary DNA (cDNA) sequence is numbered from the first base of the translation start codon and is shown in the 5′ to 3′ direction; (*) denotes the termination codon. The polyadenylation signal in 3′-Unstransleted region (UTR) is denoted in grey face. The conserved 14-3-3 domain is shaded. The direct peptide binding residues are boxed. The nuclear localization signal sequence (NLS) is shown underlined. The cDNA encodes a gene of 747 nucleotides in length and a protein of 248 amino acids in length. This sequence is submitted to the GenBank Data Bank with accession number KP099938.

The full-length cDNA of *Tm14-3-3ζ* was obtained using RT-PCR and RACE-PCR. The *Tm14-3-3ζ* cDNA comprised of 747 bp coding region flanked by 18 bp 5′-untranslated region (UTR) and a 387 bp 3′-UTR including the poly (A) tail. The deduced protein encoded by the coding region was 248 amino acids long (Figure 1). A putative polyadenylation signal (5′-ATTAAA-3′) was found 112 bp upstream of the poly (A) tail. No signal peptide was predicted in the N-terminus of the protein. Analysis of the amino acid sequence showed a 14-3-3 domain that includes the peptide binding residues (Leu-219, Ile-220, Leu-223, Asn-227, Leu-230 and Trp-231) and a nuclear export localization signal sequence (NLS) composed of thirteen amino acids (N-LIMQLLRDNLTLW-C). The full-length *Tm14-3-3ζ* sequence was deposited in GenBank under accession number KP099938.

### 3.2. Sequence Homology and Phylogenetic Analysis

The predicted amino acid sequence of Tm14-3-3ζ was found to be highly similar to other known insect 14-3-3ζ protein sequences. This was demonstrated by multiple sequence alignment of Tm14-3-3ζ amino acid sequence with NCBI retrieved sequences of other insect 14-3-3ζ proteins. The 14-3-3ζ homologues showed a high level of sequence conservation within the NLS motif of 14-3-3 domain (Figure S1A). The protein shared sequence identity that is higher than 90% with 14-3-3ζ proteins from other insect species. A sequence identity of 87% and 81% were detected with *Ceratitis capitata* and *Homo sapiens* Tm14-3-3ζ sequences, respectively. The percentage distance matrix showed a divergence of 5% to 14% in between Tm14-3-3ζ and other insect 14-3-3ζ proteins (Figure S1B). Furthermore, molecular taxonomical clustering of 14-3-3ζ proteins showed evolutionary relationships between Tm14-3-3ζ and other representative insect 14-3-3ζ proteins (Figure S1C). The phylogenetic tree showed the clustering of insect Tm14-3-3ζ proteins into their respective taxonomical orders. Importantly, Tm14-3-3ζ protein was found in a separate cluster with another coleopteran insect *Tribolium castaneum* 14-3-3ζ protein (Tc14-3-3ζ). The 14-3-3ζ proteins under the insect orders Coleoptera and Diptera form two major clusters with high bootstrap support. On the other hand, the 14-3-3ζ proteins representing Hemiptera and Lepidoptera orders showed a lower bootstrap strength. Human 14-3-3ζ (Hu14-3-3ζ) was considered as an outgroup and was used to assess the overall phylogenetic classification.

### 3.3. Developmental and Tissue Specific Expression Patterns of Tm14-3-3ζ

**Figure 2:**
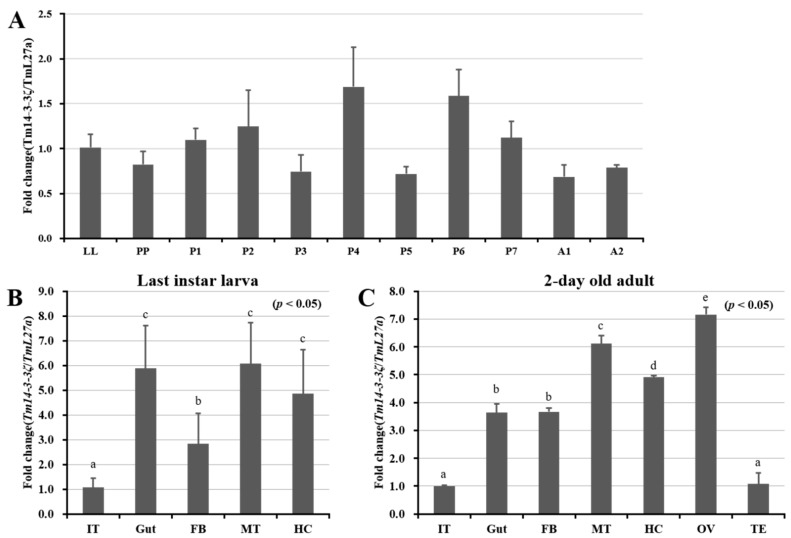
Expression analysis of Tm14-3-3*ζ* messenger RNA (mRNA) during development and in larval and adult tissues of *Tenebrio molitor*. The expression patterns were measured using quantitative polymerase chain reaction (qPCR) having synthesized cDNA samples as template. (**A**) Developmental expression patterns show the consistent expression of Tm14-3-3ζ in all stages of the life cycle; (**B**,**C**) Tissue specific expression patterns indicate higher expression of *Tm14-3-3ζ* in gut, Malpighian tubule and hemocytes of last instar larva (**B**) and Malpighian tubule and ovary of 2-day old adult (**C**). Abbreviations are as follows: *T. molitor* last instar larva (LL), Prepupa (PP), 1-day to 7-day old pupa (P1–P7) and 1-day and 2-day old adult (A1 and A2), integument (IT), fat body (FB), Malpighian tubule (MT), hemocyte (HC), ovary (OV) and testis (TE). *TmL27a* mRNA was used as an internal control. The results were statistically analyzed using one-way analysis of variance (ANOVA) and Tukey’s multiple range tests at 95% confidence levels (*p* < 0.05). Different lowercase letters represent significant differences among groups.

In order to study the expression pattern of *Tm14-3-3ζ* transcript in different developmental stages and tissues, qPCR analysis was conducted using specific primers (Table 1). *TmL27a* gene was used as an internal control for qPCR experiments. The developmental expression patterns of *Tm14-3-3ζ* was revealed using cDNAs synthesized from last instar larva, pre-pupa, pupa (pupal day 1 to day 7) and adult (day 1 and day 2). The qPCR analysis showed a consistent expression of *Tm14-3-3ζ* protein in all the developmental stages of the insect (Figure 2A). In addition, the tissue specificity of *Tm14-3-3ζ* was examined using cDNAs synthesized from gut, integument, fat body, Malpighian tubule and hemocytes isolated from last instar larva and 2-day old adults. The expression pattern of *Tm14-3-3ζ* was also analyzed in ovary and testis tissues isolated from 2-day old adults. In the last instar larval stage, higher expression of *Tm14-3-3ζ* was seen in the gut and Malpighian tubules whereas lower expression was found in the integument (Figure 2B). In 2-day old adult tissues, expression was highest in the ovaries followed by Malpighian tubules, hemocytes, gut and fat body (Figure 2C).

### 3.4. Expression Analysis of Tm14-3-3ζ in Response to Microorganisms

**Figure 3:**
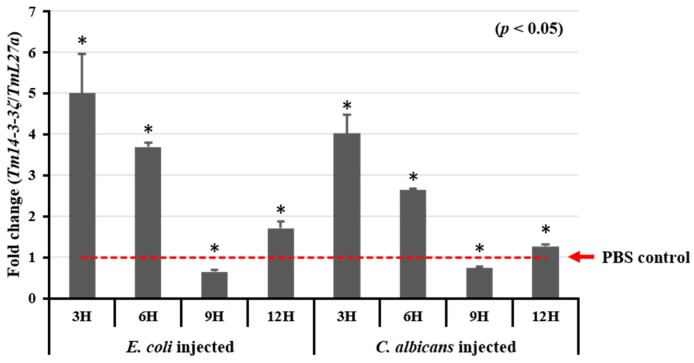
Temporal expression analysis of *Tm14-3-3ζ* after *Escherichia coli* and *Candida albicans* challenge. *E. coli* and *C. albicans* were injected into *T. molitor* larvae and samples were collected at 3, 6, 9 and 12 h post-infection. Patterns of *Tm14-3-3ζ* expression was investigated, showing the dramatic increase in *Tm14-3-3ζ* expression at 3 h post-injection of microorganisms. Results of three biological replications are presented with standard errors.

In order to identify whether *Tm14-3-3ζ* transcripts can be induced by microorganisms, the *T. molitor* last instar larvae were challenged with Gram-negative bacteria (*E. coli*) and yeast (*C. albicans*) through systemic circulation using PBS as an injection control. The expression of *Tm14-3-3ζ* increased in last instar larvae 3 h post-infection with *E. coli* and *C. albicans* (Figure 3). After an immediate upregulation of *Tm14-3-3ζ* transcripts, sudden downregulation was noticed at 6, 9 and 12 h post-infection.

### 3.5. Characterization of Polyclonal Antibody and Sub-Cellular Localization of Tm14-3-3ζ

**Figure 4:**
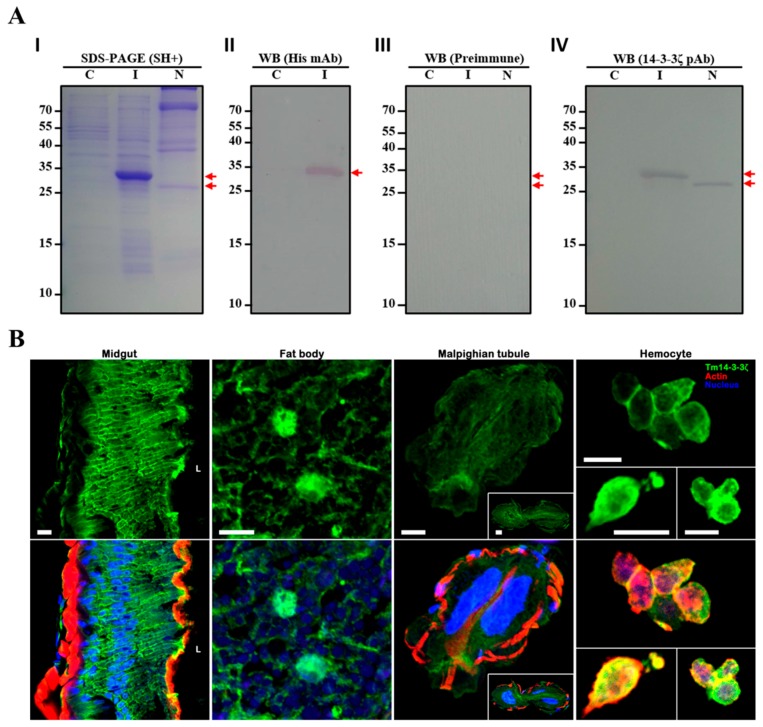
Subcellular localization of 14-3-3ζ in tissues of late instar *T. molitor* larva using anti-Tm14-3-3ζ polyclonal antiserum. Production of anti-Tm14-3-3ζ polyclonal antibody and its specificity with recombinant Tm14-3-3ζ (rTm14-3-3ζ). (**A-I**) Sodium dodecyl sulfate-polyacrylamide gel electrophoresis (SDS-PAGE) characterization of protein profiles in non-induced, isopropyl β-D-1-thiogalactopyranoside (IPTG) induced *E. coli* homogenate and late instar larval whole-body homogenate; (**A-II**) The detection of rTm14-3-3ζ using His-Tag monoclonal antibody; (**A-III**) Western blot with pre-immune blood used as negative control; (**A-IV**) Western blot for the detection of rTm14-3-3ζ and endogenous Tm14-3-3ζ using anti-Tm14-3-3ζ polyclonal antibody. C, non-induced *E. coli* homogenate; I, IPTG induced *E. coli* homogenate; N, native *T. molitor* late instar larval whole-body homogenate. (**B**) Immunohistochemical localization of the 14-3-3ζ protein in midgut, fat body, Malpighian tubules and hemocyte of late instar *T. molitor* larva. Cryosections of the harvested, fixed and washed tissues were blocked for 1 h with 2% bovine serum albumin (BSA) in phosphate buffered saline (PBS) containing Tween-20 and incubated with rabbit anti-Tm14-3-3ζ antiserum (1:300) for 3 h at 4 °C. After washing, the samples were incubated in Alexa Fluor 488 dye-conjugated secondary antibody (1:300). The immunoreactivity (green signals) of Tm14-3-3ζ localization was analyzed by an Fluoview 500 confocal microscopic system (Olympus). TO-PRO-3 Iodide and Alexa fluor 568 phalloidin was used to detect the nuclei (blue signals) and the actin (red signals) molecules.

We generated a peptide-based polyclonal antibody against Tm14-3-3ζ protein and studied its specificity with recombinant protein (rTm14-3-3ζ) using Western blot analysis. This was to study the specific immunoreactions of the polyclonal Tm14-3-3ζ antiserum against rTm14-3-3ζ and the endogenous form (Figure 4A). SDS-PAGE analysis of IPTG induced cell fractions showed a potential band corresponding to rTm14-3-3ζ. This band was found slightly higher in the separating gel compared to the endogenous form (Figure 4A-I). The rTm14-3-3ζ expressed by the IPTG-induced cells of *E. coli* (BL21-DE3 strain) was found reactive to anti-His tagged monoclonal antibody, used as the positive control (Figure 4A-II). Bands corresponding to rTm14-3-3ζ and the endogenous form of Tm14-3-3ζ were not detected in pre-immune blood, acting as the negative control (Figure 4A-III). The specificity of the Tm14-3-3ζ antiserum was validated by its ability to detect rTm14-3-3ζ as well as endogenous Tm14-3-3ζ in the protein extracts of the *T. molitor* whole body (Figure 4A-IV).

Subsequently, Tm14-3-3ζ polyclonal antibody and Alexa Flour 488 dye-conjugated secondary antibody were used to examine the localization of Tm14-3-3ζ in the gut, fat body, Malpighian tubules and hemocytes of last instar larva. The immunohistochemical analysis was performed using a confocal microscope (Figure 4B). Tm14-3-3ζ signal was found detected in the membrane and cytosol of gut and fat body. In hemocytes Tm14-3-3ζ signals were distributed in the cytosol and cell’s perimeter.

### 3.6. Tm14-3-3ζ Silencing Increases Susceptibility to Escherichia coli and Candida albicans Infection in Larvae

**Figure 5:**
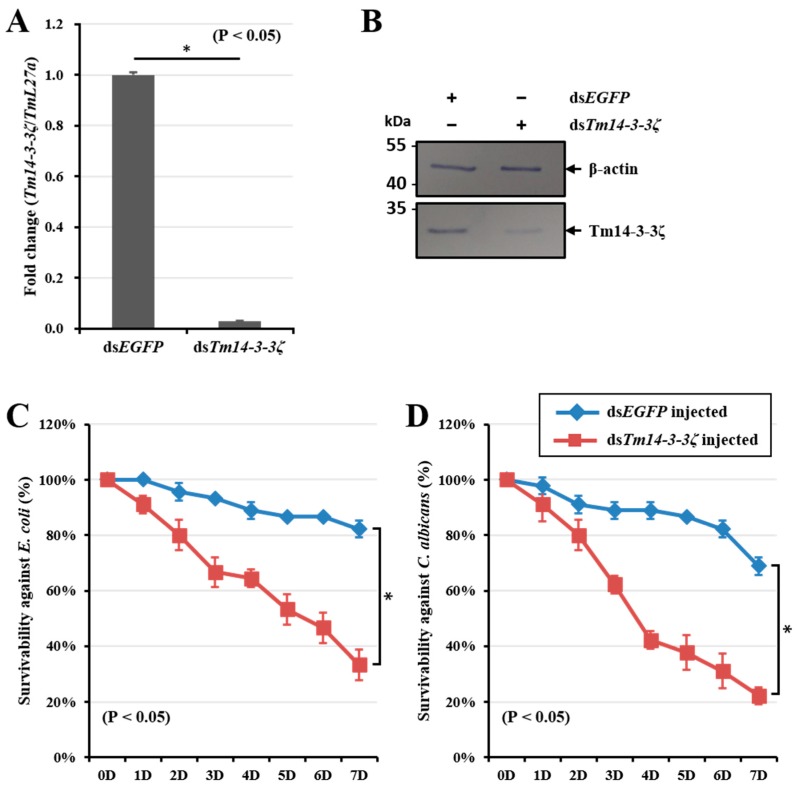
Silencing of *Tm14-3-3ζ* transcripts using RNA interference (RNAi) and mortality assay after *E. coli* and *C. albicans* injection. About 98% of *Tm14-3-3ζ* mRNA (**A**) and protein (**B**) expression were decreased by injection of 1 μg of dsTm14-3-3ζ RNA. Mortality against injection of pathogenic microorganisms such as *E. coli* (**C**) and *C. albicans* (**D**) in ds*Tm14-3-3ζ*-treated *T. molitor* larvae were investigated. The results suggest that silencing of *Tm14-3-3ζ* induces mortality in the infected larvae.

To investigate the function of *Tm14-3-3ζ* in the survival of the larvae against *E. coli* and *C. albicans* infection, we injected *Tm14-3-3ζ* dsRNA to the host larvae thereby setting a gene silencing model. The efficiency of *Tm14-3-3ζ* silencing was found to be 95-fold as compared with the ds*EGFP* injected group. The study was conducted in three biological replications and the silencing of *Tm14-3-3ζ* transcripts was found to be significant (Figure 5A). The dsRNA induced silencing of *Tm14-3-3ζ* transcript was also noticed at the protein level using Tm14-3-3ζ antiserum (Figure 5B). Subsequent *E. coli* and *C. albicans* inoculation to *Tm14-3-3ζ* silenced larvae showed a compromised immune response with a decrease in the larval survivability to almost 30% (Figure 5C) and 20% (Figure 5D), respectively. The reduced survivability of *T. molitor* larvae was significant (*p* < 0.05) at seven days post-infection of the microbes.

## 4. Discussion

The 14-3-3 family of proteins expressed in vertebrates and invertebrates are responsible for interaction with a variety of cytosolic proteins with phosphorylated serine/threonine residues and direct signaling roles [42,43]. In the vertebrate model systems, seven different 14-3-3 isoforms participate in diverse functions, while in insects two isoforms (ε and ζ) have been identified. In the coleopteran pest *T. molitor,* the functional specificity of 14-3-3 proteins remains unexplored. As previously reported, *T. molitor* is an efficient model for functional studies involving immunity, defense and physiology [28,29]. The 14-3-3ε isoform identified earlier showed hemocyte antimicrobial activity [34]. In this study, we cloned *14-3-3ζ* isoform from *T. molitor* and provide an indirect evidence for its role in phagocytosis of Gram-negative bacterium *E. coli* and yeast *C. albicans*. The putative 14-3-3 domain in 14-3-3ζ protein is responsible for regulating a wide array of cellular processes [44,45,46]. The peptide-binding residues in the 14-3-3 domain may be responsible for cross-talk of Tm14-3-3ζ with other interacting proteins and channelizing regulatory processes [47]. Multiple alignments of Tm14-3-3ζ with 14-3-3ε isoform of *T. molitor* show 14-3-3 conserved domain and residues including the NLS sequence. In light of high level of sequence similarity of Tm14-3-3ζ to 14-3-3ζ proteins in other species, which have a role in phagocytosis, it is likely that the protein in *T. molitor* is playing a similar role. In the planarian, *Dugesia japonica*, the 14-3-3ζ and 14-3-3ε possess conserved domains with a common phosphorylated site [48]. The sequence identity of the 14-3-3 isoforms could reflect functional redundancy and the minor sequence variations might be responsible for their differential binding activity [49]. As reported earlier for the 14-3-3ε/ζ isoforms, the essential serine residues for interactions with different ligands are conserved [50]. These conserved serine residues were found at 187th and 233rd position in Tm14-3-3ζ. Another striking identity in the sequence conservation of 14-3-3ζ proteins is the annexin-like consensus sequence (N-MKGDYYRYLAEVTRNAVV-C) in the 14-3-3 domain, which may have functional importance during the 14-3-3 mediated exocytosis [51]. With regards to immunological functions, it is found that the 14-3-3ε transcript from *T. molitor* is responsible for a decreased secretion of antimicrobial peptides (AMPs) from hemocyte to hemolymph [34], while Tm14-3-3ζ is responsible for phagocytosis function against *E. coli* and *C. albicans*. In any case, silencing of both the transcripts leads to reduced survivability of the larvae after microorganism inoculation.

The *Tm14-3-3ζ* transcript was found consistently throughout the developmental stages and in different tissues of the insect. In *Aedes aegypti* the *14-3-3ζ* isoforms were identified in the larval and pupal stages during the mosquito development. The transcripts were also expressed in the head, ovary, fat body and midgut tissues of adult female mosquitoes [52]. Among the ε and ζ isoforms of 14-3-3 proteins, the *14-3-3ζ* isoform generally shows markedly higher expressions in all stages and tissues in comparison with the *14-3-3*ε isoform. This has been found to be true in the *B. mori* [16] and *A. aegypti* 14-3-3ε/ζ isoforms [52]. The expression of Tm14*-3-3ζ* transcripts showed a sudden increase followed by a sharp decline after inoculation of the insect larvae with *E. coli* and *C. albicans*. It has been reported that the stimulation by pathogen associated molecular patterns such as lipopolysaccharide (LPS), peptidoglycan (PGN), teichoic acids and so forth could lead to expression of *Tm14-3-3ζ* transcript with immune-related functions [53]. The 14-3-3ζ proteins promote immunity by regulating TLR-3 signaling through TICAM-1 pathway [54] and immune responses through Stat3 signaling in oral cancers [55]. The 14-3-3ζ proteins promote phagocytosis and microbial clearance in *Drosophila* hemocytes. This has been demonstrated through in vivo phagocytosis assay involving 14-3-3ζ screened from RNA interference (RNAi)-based phenotypic screens [23].

rTm14-3-3ζ was successfully expressed in the *E. coli* system. The rTm14-3-3ζ and the endogenous Tm14-3-3ζ reacted with the Tm14-3-3ζ antiserum that was synthesized commercially from a 16-mer region at the C-terminal end. Our findings highlight protein bands at ~35 kDa and ~27 kDa corresponding to rTm14-3-3ζ and the endogenous form, respectively. The Tm14-3-3ζ antiserum was then utilized for the immunohistochemical localization of Tm14-3-3ζ proteins in tissues of *T. molitor* larvae. The subcellular localization of Tm14-3-3ζ protein in all the major tissues including gut, fat body, Malpighian tubules and hemocytes were investigated. The results are consistent to an earlier report of sub-cellular localization of *14-3-3ζ* gene in the Indian meal moth, *Plodia interpunctella* [21]. The immunohistochemical observations in the present study show extensive localization of Tm14-3-3ζ proteins in the cytosolic space of the larval tissues. In the gut and fat body, Tm14-3-3ζ was localized on the membrane and in the cytosol, respectively. Like other members of the 14-3-3 family, Tm14-3-3ζ was found distributed in the cytosol and selectively along the membrane of some cells. 14-3-3ζ has been found in the mitochondria, microsomes and nuclear compartments of the mouse hippocampus [56,57]. The *Opisthorchis viverrini* 14-3-3ζ isoform were expressed throughout the tissues except in the gut epithelium and expression was highest in the testis, suggesting a potential role in spermatogenesis [58].

RNAi-based functional analysis has been useful for the characterization of several immune-related genes such as *MyD88*, *CD63*, *apolipophorin-III* and *PGRP-LE* in response to microorganism challenge [28,30,31,33,59]. Recently, the Tm14-3-3ε isoform was found to play a role in secretion of antimicrobial peptide in response to *E. coli* [34]. Further, 14-3-3ζ isoform is a novel candidate gene that promotes phagocytosis in *Drosophila* model and is required for the survival of host against the Gram-positive bacterium, *Staphylococcus aureus* [23]. In the same light, a functional role could be attributed to Tm14-3-3ζ eliciting innate immune responses since silencing of 14-3-3ζ transcripts in the larvae resulted in a reduction in larval survivability against *E. coli* and *C. albicans.* An earlier report demonstrated that 14-3-3ε silencing in *T. molitor* impairs the exocytosis of AMPs from hemocyte to hemolymph and affects the bactericidal action on the microbes [34]. In the context of the present study, Tm14-3-3ζ is critical for larval survivability against normal bacterial and fungal infections. In addition, established studies suggest that 14-3-3ζ play an important regulatory role in apoptosis [1,5], autophagy [2,57,60] and phagocytosis of microbes [23]. Similar experiments would be required to confirm the role of Tm14-3-3ζ more precisely. A better understanding of Tm14-3-3ζ role in innate immunity of *T. molitor* will be a critical towards its bio-control in agricultural fields and helping in improve the regulation of critical biological processes in the insect.

## 5. Conclusions

We have screened and characterized a novel isoform of 14-3-3 family from the coleopteran pest *T. molitor* EST and RNA Sequencing database (Tm14-3-3ζ) and generated a peptide-based polyclonal antibody and validated it against the endogenous and recombinant Tm14-3-3ζ. Finally, we have characterized functional role of Tm14-3-3ζ in larval survivability against *E. coli* and *C. albicans* using RNAi technique in *T. molitor.*

## Figures and Tables

**Table 1:** The primers used in this study.

Name	Primer Sequences
Tm14-3-3ζ 5′-RACE GSP1	5′-GATGAGAGTGCTGTCCTT-3′
Tm14-3-3ζ 5′-RACE nGSP2	5′-TAATCTGATGGGATGTGTGGGCGT-3′
Tm14-3-3ζ 3′-RACE GSP1	5′-ATTTGATCCCCAAAGCAAGC-3′
Tm14-3-3ζ 3′-RACE nGSP2	5′-CCCACACATCCCATCAGATT-3′
Tm14-3-3ζ Ex(*Bam*HI) Fw	5′-GGGGGATCCATGTCCGTCACTGACAAAGATGAG-3′
Tm14-3-3ζ Ex(*Hind*III) Rv	5′-GGGAAGCTTCAATTATCACCAGTCTCTTGAGGC-3′
Tm14-3-3ζ qPCR Fw	5′-TTTGGCGGAAGTAGCCACAGGAGA-3′
Tm14-3-3ζ qPCR Rv	5′-TAATCTGATGGGATGTGTGGGCGT-3′
TmL27a qPCR Fw	5′-TCATCCTGAAGGCAAAGCTCCAGT-3′
TmL27a qPCR Rv	5′-AGGTTGGTTAGGCAGGCACCTTTA-3′
*Bam*HI and *Hind*III recognition sites have been underlined.	

RACE: Rapid amplification of cDNA ends; qPCR: Quantitative polymerase chain reaction.

## Supplementary Materials

The following are available online at http://www.mdpi.com/2073-4425/9/7/330/s1, Figure S1: Molecular phylogenetic and percent identity analysis of 14-3-3ζ isoform in *T. molitor*, Table S1. Gene information used.
