# Supplementary figures and images for: Molecular Cloning and Effects of Tm14-3-3ζ-Silencing on Larval Survivability Against E. coli and C. albicans in Tenebrio molitor
Source: Genes (Basel). 2018 Jun 29;9(7):330. doi: 10.3390/genes9070330 (PMC6070784; doi:10.3390/genes9070330)

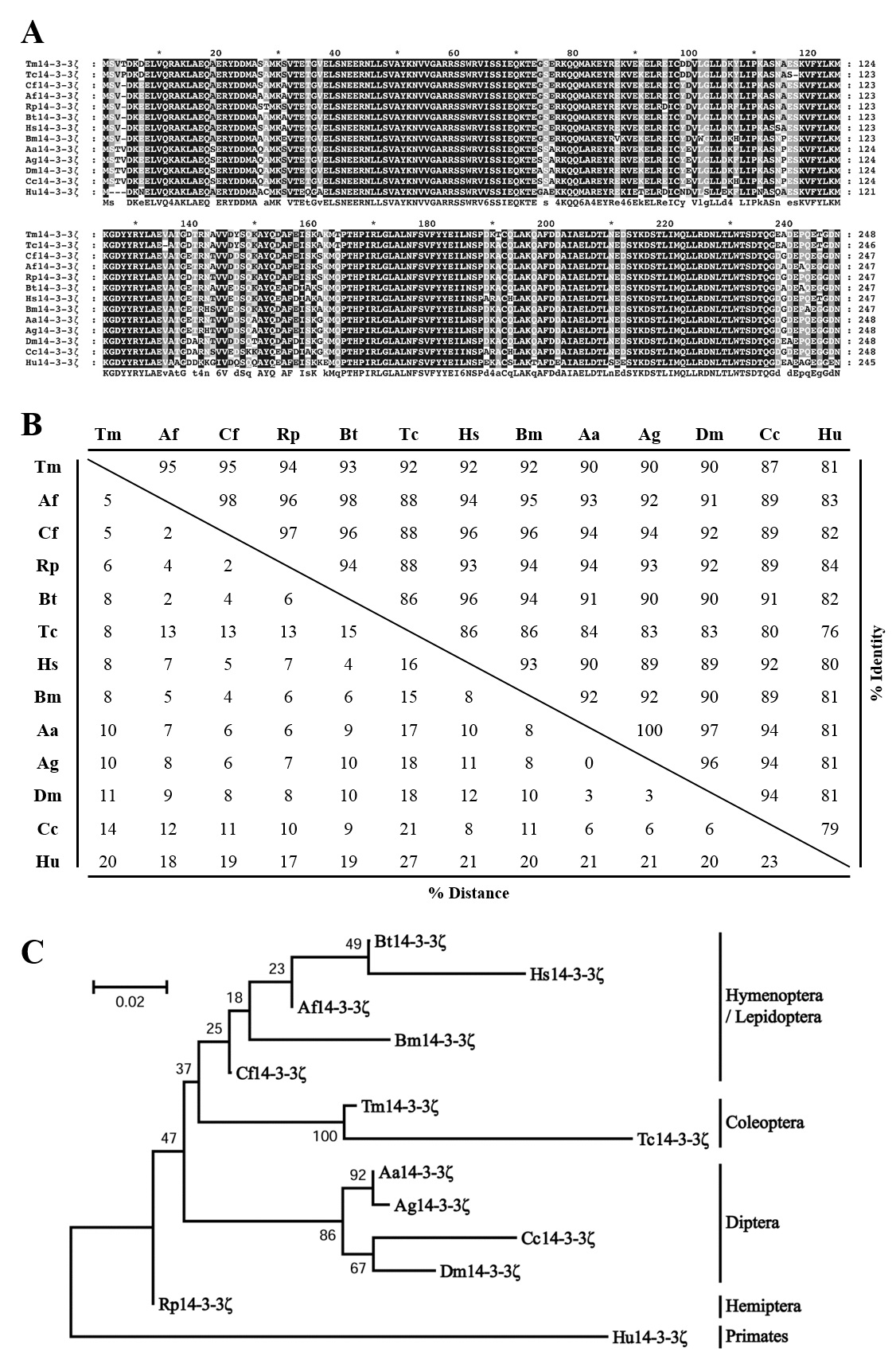

Supplement: Supplementary file 1 [file genes-09-00330-s001.jpg]
